# Dietary diversity among pregnant women and associated factors in Ethiopia: Systematic review and meta-analysis

**DOI:** 10.1371/journal.pone.0251906

**Published:** 2021-06-10

**Authors:** Abebaw Gedef Azene, Abiba Mihret Aragaw, Habtamu Tilaye Wubetie, Gizachew Tadesse Wassie, Gebiyaw Wudie Tsegaye, Muluwork Ayele Derebe, Habitamu Dessie Mitiku

**Affiliations:** 1 Department of Epidemiology and Biostatistics, College of Medicine and Health Science, Bahir Dar University, Bahir Dar, Ethiopia; 2 Department of Statistics, College of Natural and Computational Sciences, Debre Markos University, Debre Markos, Ethiopia; 3 Department of Statistics, College of Natural and Computational Sciences, University of Gondar, Gondar, Ethiopia; 4 Department of Statistics, College of Sciences, Bahir Dar University, Bahir Dar, Ethiopia; 5 Department of Statistics, College of Natural and Computational Sciences, Injibara University, Injibara, Ethiopia; Universitas Airlangga, INDONESIA

## Abstract

**Backgrounds:**

Pregnancy related complications are major causes of maternal morbidity and mortality worldwide. Diversified food consumption is essential to produce hormones during pregnancy and it reduced complications. In Ethiopia, many researchers were investigated about the proportion of pregnant women with dietary diversity and its determinant factors. However, those studies are inconsistent and fragmented. Therefore, the aim of this study was to estimate the pooled proportion of pregnant women with dietary diversity practice and its associated factors in Ethiopia.

**Methods:**

We conducted a systematic electronic web-based search of PubMed/ /MEDLINE, EMBASE, Web of Science, Google Scholar and Google online databases for identifying studies on proportion of pregnant women with dietary diversity practice and its associated factors in Ethiopia using pre-defined quality and inclusion criteria. STATA version 14 statistical software was used to analyze the data. We extracted relevant data and presented in tabular form. The I^2^ test was used to assess heterogeneity across studies. Funnel plot asymmetry and Begg’s test were used to check for publication bias. The final effect size was determined by applying a random-effects model.

**Results:**

Our search identified 170 studies. Of which, 23 were included in the final analysis stage. The pooled proportion of dietary diversity among pregnant women in Ethiopia was 41% (95% CI: 33, 49). Mothers can read and write (OR = 1.82 (95% CI: 1.25, 2.64)), maternal primary school and above educated (OR = 2.11 (95% CI: 1.10, 4.05)), nutritional information (OR = 4.1 (95% CI: 2.1, 7.99), dietary diversity knowledge (OR = 3.4 (95% CI: 2.73, 4.73)) and household had rich wealth index (OR = 3.45 (95% CI: 1.19, 10.1)) were significantly associated with dietary diversity practice during pregnancy.

**Conclusions:**

In this meta-analysis; we found that low proportion of pregnant women with adequate dietary diversity in Ethiopia (41%). Maternal education, nutritional information, dietary diversity knowledge and wealth index level of household were significantly associated factors of pregnant woman with dietary diversity practice. This finding implies that improving the awareness of woman about dietary diversity during pregnancy and empowering women economically would play a significant role to improve dietary diversity practice.

## Background

Pregnancy related complications are a major public health problem in the world. To reduce it, diversified food consumption is essential during pregnancy. Dietary diversity intake fulfills the requirements of minerals and vitamins of a pregnant woman and the growing of the fetus. For healthy development of fetus taking Minerals and Vitamins are important. Dietary diversity is the consumption of a variety of food which is nutritionally adequate over a reference period [1–3].

Researchers suggested that nutrition information, education, monthly income, dietary knowledge, family size, age, ownership of radio, having an illness, and husband occupation as factors those affect the dietary practices of pregnant women [4–7].

The burden of inadequate dietary diversity intake among pregnant women is double which leads to poor fetus development, and increases pregnancy related complications [8]. Savy M et al (2007) indicated that maternal mortality, low birth weight, and childhood stunting are the major consequences of low diversified food intake during pregnancy [3]. Globally, 99% of maternal deaths are in developing countries, and most of them were related with inadequate food diversity intake and poor nutrition [9].

Ethiopia is one of the developing countries with high maternal mortality related with pregnancy complication. Annually, more than 10% pregnancies end in abortion, and 1 in 27 mothers die due to complications of pregnancy or childbirth [10]. By 2025, WHO planned to reduce low birth weight by 30% [11, 12], to fulfill this agenda, it needs to assess the status of dietary diversity intake among pregnant women during pregnancy [13]. The proportion of pregnant women with dietary diversity in Ethiopia scaled from 12.8 to 74.5% [14, 15].

In Ethiopia, many researchers were investigated about the proportion of pregnant women with dietary diversity and its determinant factors. However, those studies are inconsistent and fragmented. Hence, our concern was to have a comprehensive national estimate for the overall proportion of pregnant women with adequate dietary diversity and its associated factors. Therefore, the aim of this study was to estimate the pooled proportion of pregnant women with dietary diversity practice and its associated factors in Ethiopia. The findings of this study intended to improve the interventions of health care workers in dietary diversity of women during pregnancy, and to remained researchers for the future.

## Methods

### Search strategy and studies identification

Articles reviewed in this systematic review and meta-analysis was accessed through electronic web-based database searches, and reference list reviews. It is in accordance with the Preferred Reporting Items of Systematic Reviews and Meta-Analysis (PRISMA) protocols checklist guidelines. A comprehensive search was done from PubMed/ /MEDLINE, EMBASE, web of Science, Google Scholar and Google electronic international databases. We searched combining MeSH terms such as: “dietary diversity”, “pregnancy”, “associated or determinant factors” and “Ethiopia”. These search terms have pre-defined to allow a comprehensive search strategy that included all fields within records and Medical Subject Headings (MeSH terms). This study also used Boolean operator (within each axis we combined keywords with the "OR" operator and we then linked the search strategies for the two axes with the "AND" operator) to search dietary diversity. Searches were searched between January 1, 2020 and February 18, 2020. All articles done until April 20, 2020 were included. After identifying potentially relevant articles, studies were retrieved and managed using Endnote X8.

### Eligibility criteria

In this systematic review and meta-analysis, studies were eligible based on the following criteria; 1. Only studies conducted in Ethiopia. 2. Only studies written in English language. 3. All observational (cohort, case-control and cross-sectional) studies conducted among pregnant woman. 4. Only studies estimated proportion or associated factors of dietary diversity practice. 5. Only published studies done until April 20, 2020 were included.

### Outcome variable

Dietary diversity is the variety of foods which consumed by pregnant women out of the ten food groups. These food groups include starch staples, vitamin A-rich vegetables and fruits; dark green leafy v1egetables; other vegetables; other fruits; meat, poultry, and fish; eggs and pulses/legumes; nuts and seeds; and dairy products [16]. A good dietary diversity is considered a woman consumes five or more variety of food of the ten food groups used in this study. As a result, a primary outcome of this study was the proportion of mothers with dietary diversity during pregnancy, which was calculated as the number of women who experienced good dietary diversity during pregnancy among all pregnant women. The second outcome was associated factors conducted in Ethiopia. Odds ratio/ 2x2 contingency were extracted for associated factors.

### Data extraction

The authors used two stages of screening. Primarily, we screened the titles and abstracts based on the inclusion criteria. Secondly, we identified potentially relevant articles using titles and abstracts for further re-screening of its full article document. The relevance of the articles was evaluated based on their topic, objectives, and methodology as listed in the abstract. The abstracts were also assessed for agreement with the inclusion criteria. When it was unclear whether an abstract was relevant, it was included for retrieval. Two authors (AG and AM) extracted all essential data independently. Data were extracted using predefined format. The format contains, primary author name, year of publication, regions where the study was conducted, study area, sample size, study design, proportion (%), and response rate (%). Furthermore, contingency tables were extracted from studies which reported associated factors. Any disagreement between the two authors due to inclusion and data collection was solved by third and fourth author (GT and GW) through discussion.

### Risk of bias assessment

The risk of bias for the included studies were assessed using the 10-item rating scale developed by Hoy et al. for observational studies [17]. Sampling, data collection, reliability and validity of study tools, case definition, and study periods were assessed. Researchers categorized each study as having a low risk of bias (“yes” answers to domain questions) or high risk of bias (“no” answers to domain questions). Each study was assigned a score of 1 (Yes) or 0 (No) for each domain, and these domain scores were summed to provide an overall study quality score. Scores of 8–10 were considered as having a “low risk of bias”, 6–7 a “moderate risk”, and 0–5 a “high risk”. Two independent reviewers (AG and HD) critically appraised each paper. Disagreements between those reviewers were solved by (GT and GW) through discussion. If not, a third and fourth reviewer was involved to resolve the inconsistencies between the two independent reviewers. For the final risk of bias classification, discrepancies between the reviewers were resolved via consensus.

### Data processing and analysis

After extracted all essential data, it was entered into Microsoft Excel, and then exported to STATA version 1 Software to determine the proportion of pregnant mothers with dietary diversity. Heterogeneity across studies was assessed using the inverse variance (I^2^) and Cochran Q statistics with 25% as low, 50% as moderate, and 75% as severe heterogeneity [18]. The value of I^2^ greater than 75 was considered as the existence of sever heterogeneity in the studies [19, 20]. Dersimonian and Liard random effect model was used for analysis due to high heterogeneity [21, 22]. Subgroup analysis were conducted using region, study design and sample size [23]. As a result, we used forest plot to show the findings. We used funnel plot asymmetry, and Egger’s and Begg’s test to check for publication bias [24].

## Results

**Fig 1:**
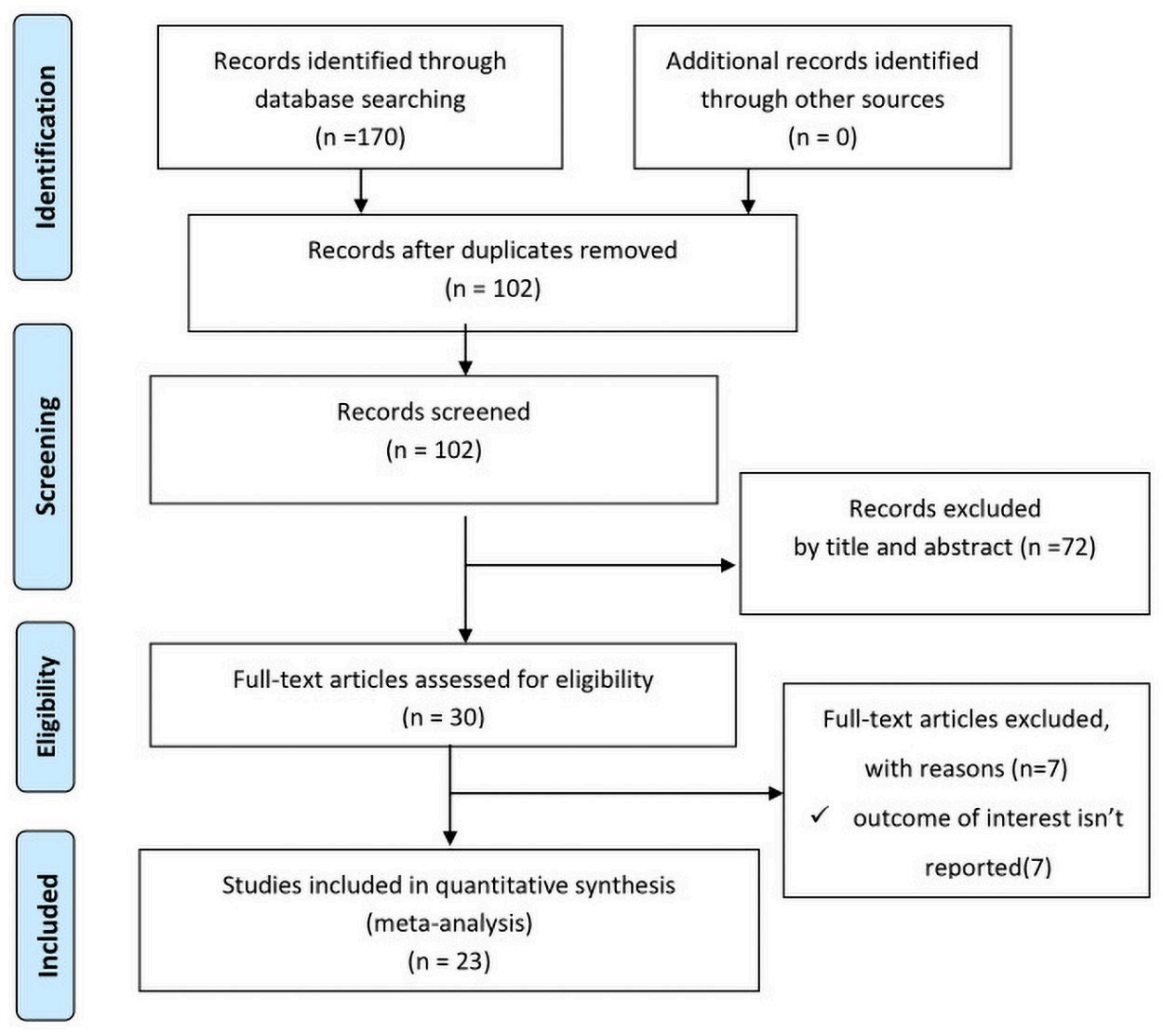
PRISMA flowchart. Flow diagram of studies included in meta-analysis.

The database search and desk review yielded a total of 170 articles from the above listed electronic sources. After reviewing the titles and abstracts, we excluded 68 **articles** due to duplication. Seventy two articles were also excluded with a reason of irrelevance and seven additional articles were excluded due to lack of response on the outcome of interest [25–32]. Finally, 23 studies were included in this systematic review and meta-analysis [5–7, 14, 15, 33–50] (Fig 1).

### Characteristics of included studies

Detail descriptions of the included studies were presented in Table 1. All studies were published between 2013 and 2020. Of all the included studies, nine studies [5, 7, 14, 33–35, 38, 40, 49] were community based cross-sectional (CBCS) and the rest; fourteen studies were institution based cross-sectional (IBCS) studies study design. Out of 23 studies, only 12 studies [5, 7, 14, 34–36, 38, 39, 42, 44, 48, 49] were reported essential associated factors of dietary diversity among pregnant woman in Ethiopia. The sample sizes in each study varies from 104 [51] to 1393 [15].

**Table 1:** Descriptive summary of 23 studies included in meta-analysis of dietary diversity during pregnancy in Ethiopia.

Author name	Publication year	Region	Study design	Sample size	Proportion (%)	Response rate (%)
Abebe, et al [33]	2014	SNNP	CBCS	104	50.0	
Alemayehu, et al [34]	2016	Amhara	CBCS	574	40.1	98.9
Aliwo, et al [35]	2019	Amhara	CBCS	647	31.4	97.4
Demilew, et al [38]	2020	Amhara	CBCS	694	19.9	97.5
Desta, et al [39]	2019	Oromia	IBCS	315	25.4	95.7
Diddana [14]	2019	Amhara	CBCS	604	74.5	100
Daba, et al [36]	2013	Oromia	IBCS	419	47.0	99.3
Hailu, et al [42]	2019	Oromia	IBCS	422	44.8	99.7
Jemal, et al [44]	2019	Tigray	IBCS	412	61.2	98
Nana, et al [5]	2018	Amhara	CBCS	616	39.3	100
Hailu1, et al [43]	2020	Oromia	IBCS	419	33.9	99.5
Tenaw, et al [6]	2018	A.A	IBCS	322	34.5	94.7
Tolera, et al [7]	2018	Oromia	CBCS	338	25.0	98.5
Yeneabat, et al [49]	2019	Amhara	CBCS	759	44.3	91
Zerfu, et al [50]	2019	Oromia	IBCS	432	49.7	100
Samuel, et al [46]	2020	SNNP	IBCS	423	20.1	97
Gebreselassie, et al [40]	2013	SNNP	CBCS	700	65.3	93.3
Workicho, et al [15]	2018	Oromia	IBCS	1393	12.8	
Shenka, et al [47]	2018	Driedewa	IBCS	380	43.0	98.2
Tefera, et al [48]	2020	A.A	IBCS	402	60.9	99
Kobiro, et al [45]	2020	SNNP	IBCS	303	42.6	99
Gizahewu, et al [41]	2019	SNNP	IBCS	211	31.8	100
Delil, et al [37]	2018	SNNP	IBCS	314	47.9	98.4

Moreover, four Ethiopian regions and two administrative cities were represented. Seven studies were in Oromia regions, six studies in Amhara region, six studies in South Nation Nationality People (SNNP) region, two studies in Addis Ababa (A.A), one study in Drie Dewa city and one study in Tigray. Furthermore, the proportion of dietary diversity varies from 12.8% [15] to 74.5% [14]. The smallest proportion was conducted institution based cross-sectional study, which was reported in Oromia region [15], while the largest was conducted community based cross-sectional study, which observed in Amhara region [14], (See Table 1).

### Risk of bias assessment of included studies

In this review, we assessed the risk of bias for each of the original studies using Hoy, et al (2012) risk-of-bias assessment tool (S1 Table). Of the total included studies, our summary assessment showed that one (4.35%) study was at high risk of bias, 3 (13.04%) were medium risk, whereas the rest 19 (82.61%) were low risk of bias.

### Proportion of dietary diversity among pregnant women

**Fig 2:**
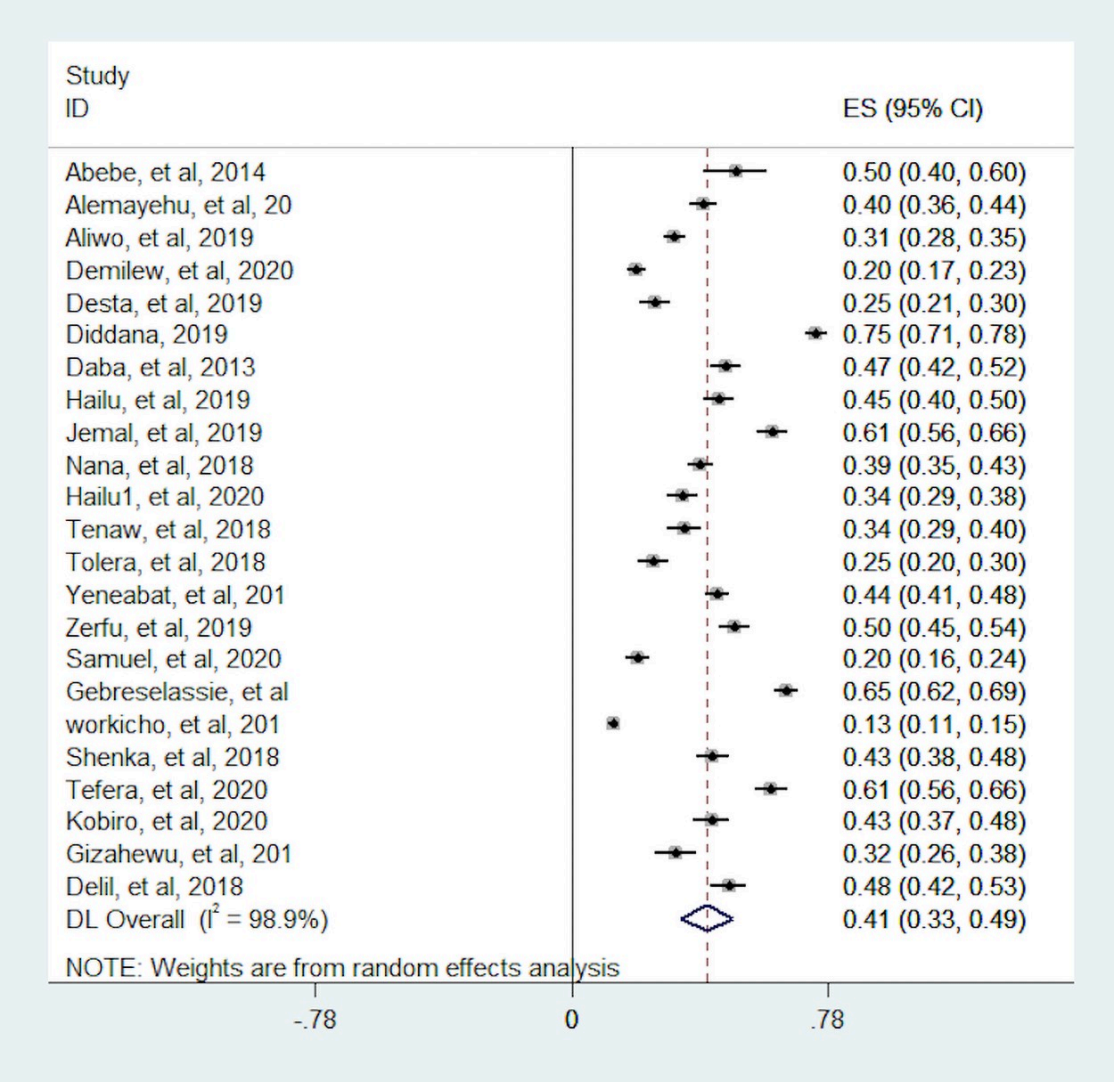
Forest plot the proportion of women with dietary diversity in Ethiopia.

**Fig 3:**
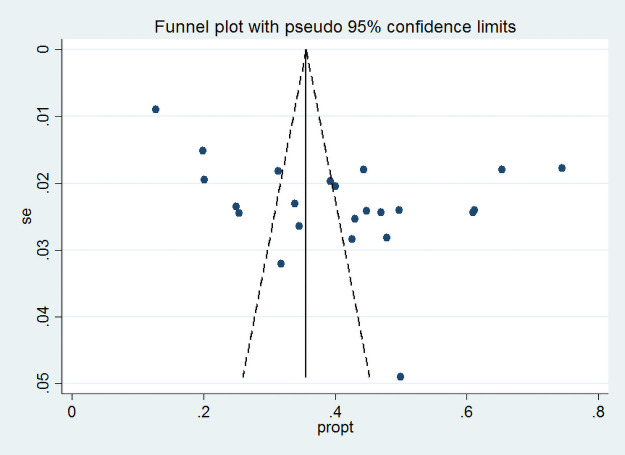
Funnel plot for the proportion of adequate dietary diversity in Ethiopia.

In this meta-analysis, we observed that the proportion of pregnant women with dietary diversity was varying across the included studies. The proportion was varying between 12.8% (15) and 74.5% (14). The I^2^ consistence test and Cochrane Q heterogeneity test statistics showed high heterogeneity (I^2^ = 98.9, p-value <0.001), which leads us Dersimonian and Liard random effect model. As a result of a random effect model, the pooled proportion of dietary diversity among pregnant women in Ethiopia was 41% (95% CI: 33, 49) (Fig 2). The funnel plot seems asymmetry but Begg’s rank correlation publication bias test was insignificant (p = 0.75) (Fig 3).

#### Subgroup analysis

**Fig 4:**
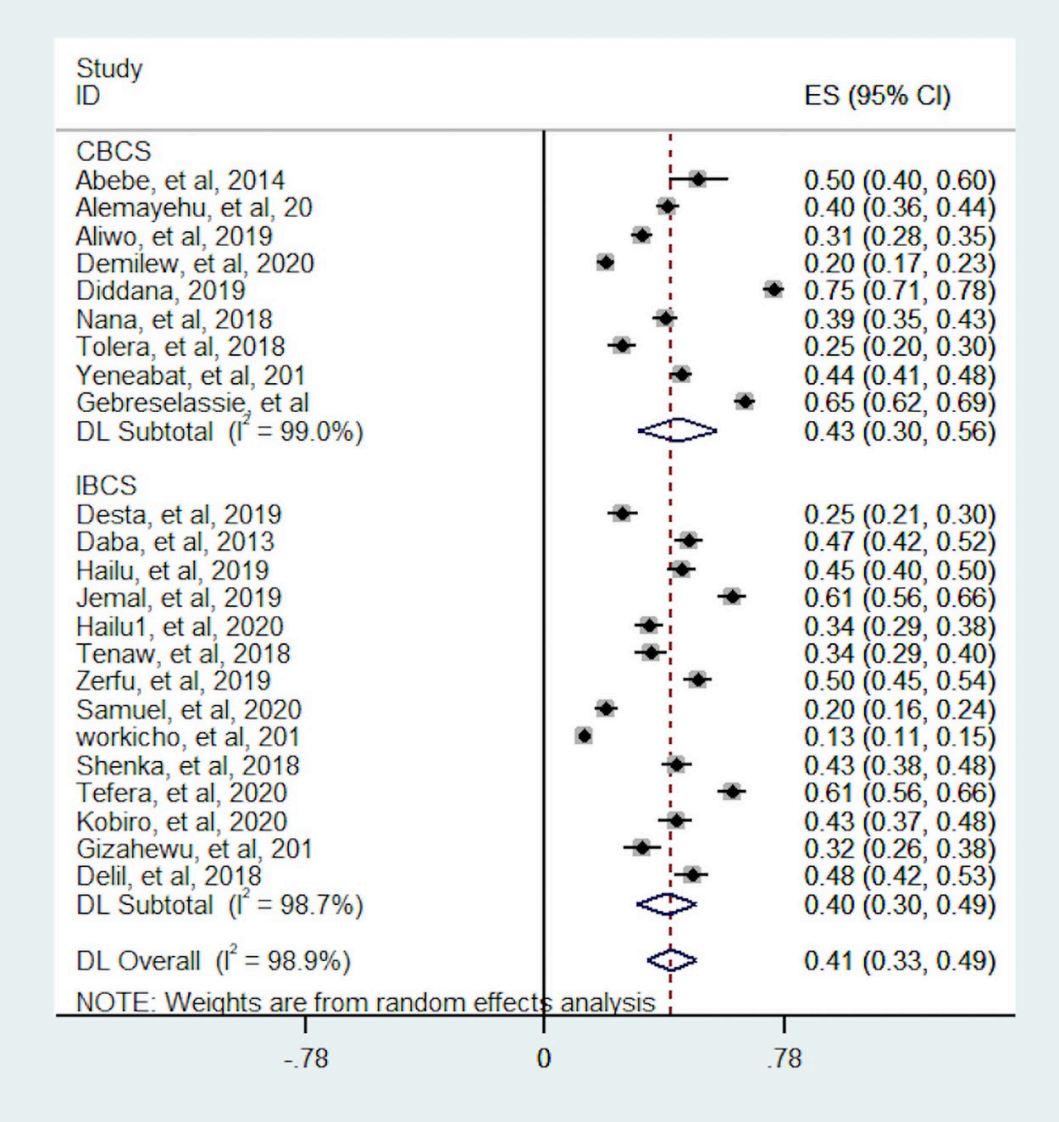
Subgroup analysis of the proportion of pregnant women with dietary diversity by study design. CBCS = Community based cross-sectional, IBCS = Institutional based cross-sectional.

**Fig 5:**
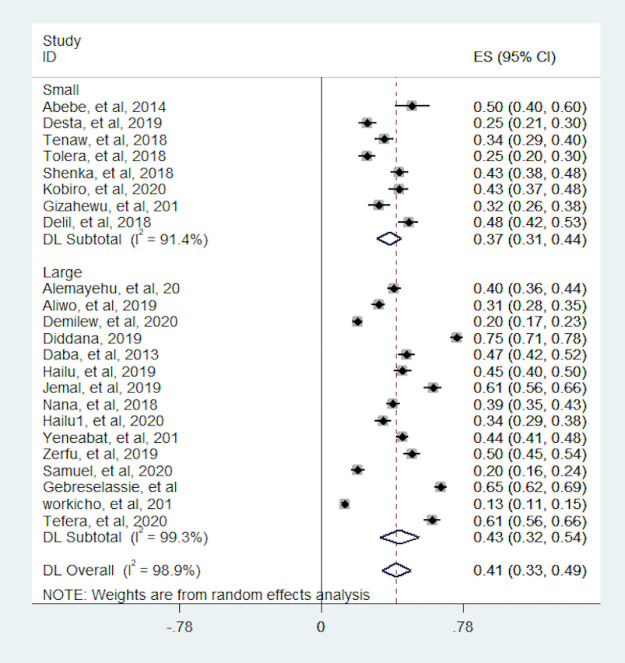
Subgroup analysis of the proportion of pregnant women with dietary diversity by sample size. Small = < = 400, large = >400.

**Fig 6:**
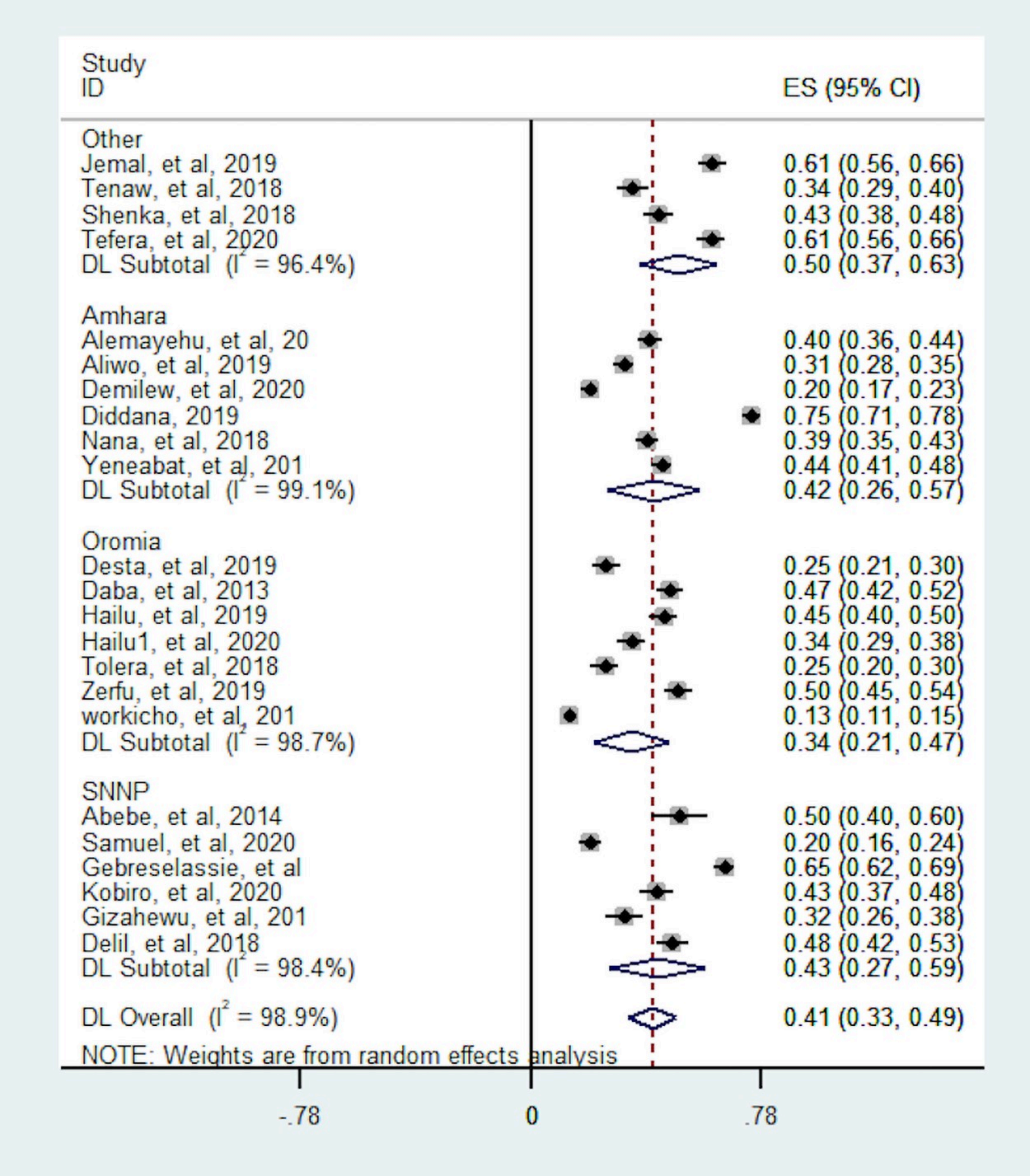
Subgroup analysis of the proportion of pregnant women with dietary diversity by region. Other = Addis Ababa, Drie Dewa and Tigray.

In this meta-analysis, we conducted subgroup analyses to identify potential source of heterogeneity and the distribution of dietary diversity based on study design, region of the country where the study was conducted and sample size. As a result; the proportion of pregnant women with dietary diversity was higher for studies which conducted with CBCS design (43.3%) than IBCS (39.7%). Heterogeneity test between study design was statistically significant (p-value <0.001). The proportion of dietary diversity were more varies in studies which conducted in CBCS and which ranged from 20% to 75% whereas studies which was conducted in IBCS the proportion relatively consistent ranged from 13% to 61% (Fig 4). The subgroup analyses based on sample size showed the pooled proportion is robust over sample size. The heterogeneity test based on sample size also statistically insignificant (p-value = 0.970) (Fig 5). Moreover, the highest pooled proportion of pregnant women with dietary diversity was 49.9%, which was observed in other group (A.A, Dreie Dewa and Tigray) and the smallest pooled proportion was 34%, which was observed in Oromia region. We found a significant heterogeneity difference between regions (p-value <0.001). The heterogeneity of within studies in Amahara and SNNP region was high which may relate to study design (Fig 6).

### Associated factors of dietary diversity among pregnant women

In this meta-analysis, we assessed factors associated with dietary diversity. A separate analysis was conducted for each factor, which was considered in this study. Variables which assessed with the dietary diversity were: maternal education, husband education, place of residence, family size, nutritional information, dietary diversity related knowledge, income, and wealth index.

#### Maternal education

**Fig 7:**
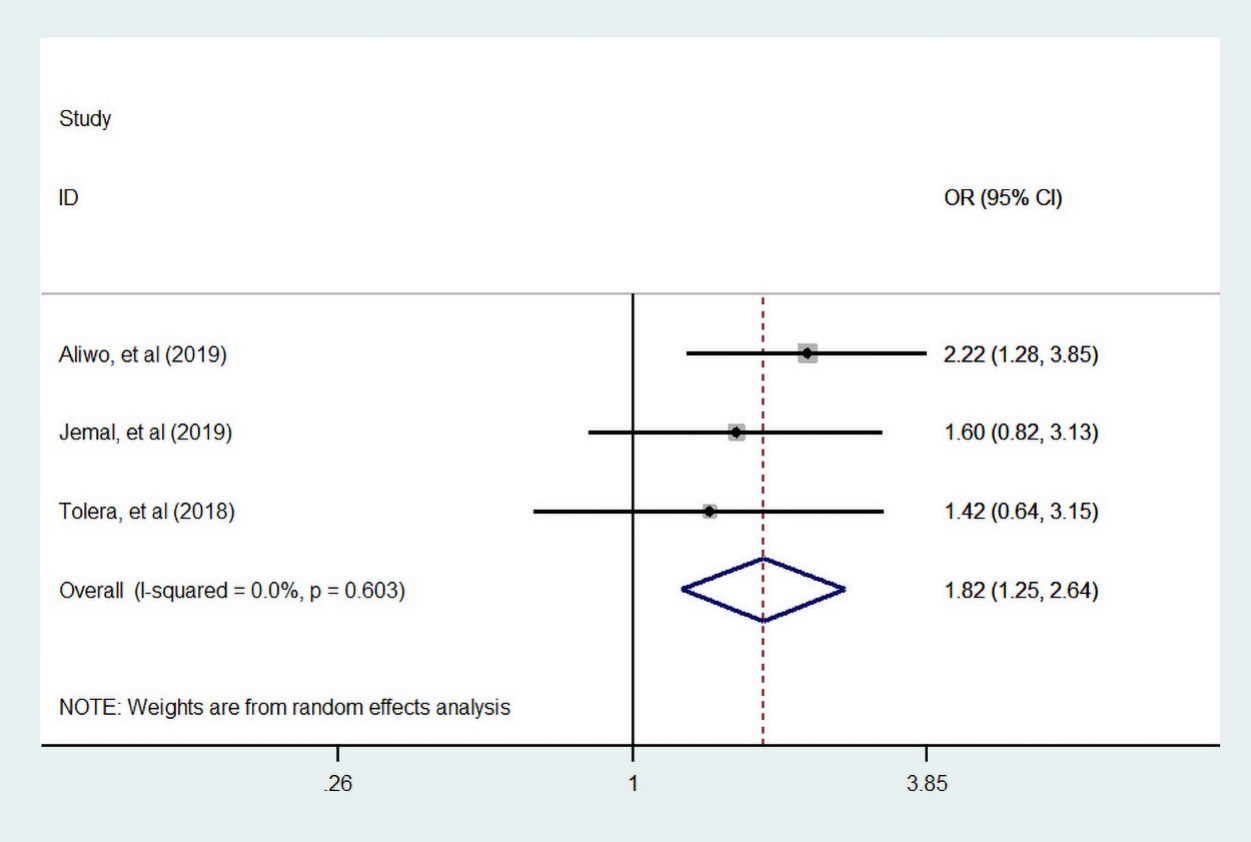
The pooled odds ratio of the association between maternal can read and write and dietary diversity during pregnancy.

**Fig 8:**
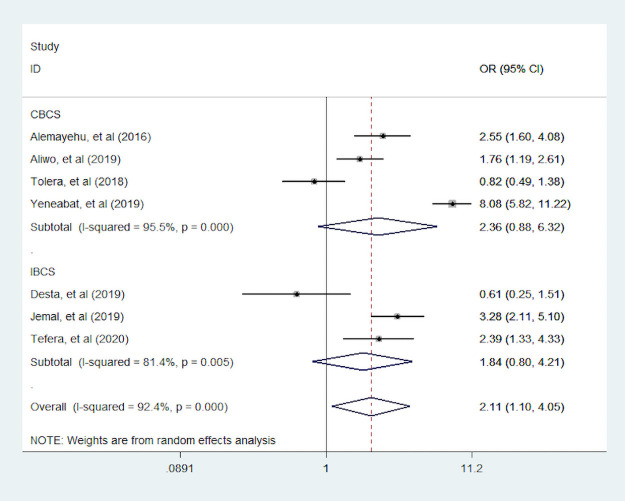
The pooled odds ratio of the association between maternal educated primary school and above with dietary diversity during pregnancy.

Three studies [7, 35, 44] assessed the association of a woman who can read and write with dietary diversity during pregnancy. Women who can read and write were 82% more likely to consumed diversified food than who cannot read and write (Fig 7). Seven studies [7, 34, 35, 39, 44, 48, 49] were assessed the primary school educated with dietary diversity. Of those seven studies, two studies reported the association between primary school educated women with dietary diversity during pregnancy is insignificant. In addition, four studies were conducted by CBCS study design. We found pregnant woman who are educated primary school and above were 2.11 times more likely to eat dietary diversity food compared to no educated women. The overall and within study design the value of I ^2^ was high, this heterogeneity may come do to study design variation (Fig 8).

#### Husband education

**Fig 9:**
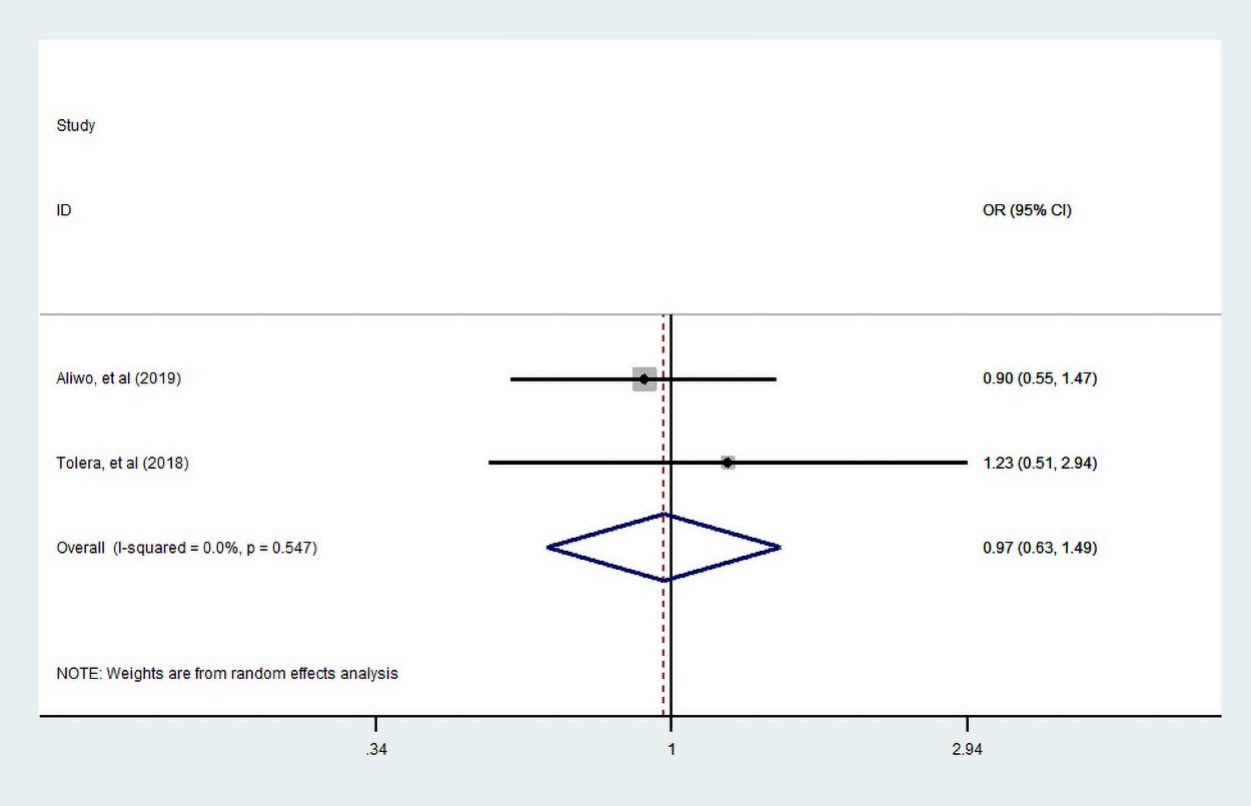
The pooled odds ratio of the association between husbands can read and write with dietary diversity during pregnancy.

**Fig 10:**
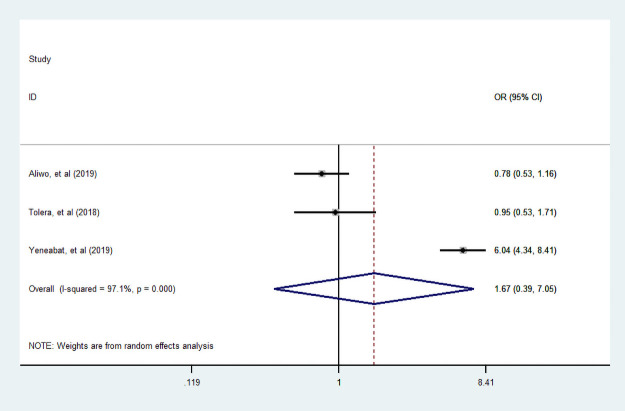
The pooled odds ratio of the association between husband educated primary school and above with dietary diversity during pregnancy.

Of three studies [7, 35, 49] assessed the association between husband education and dietary diversity practice, only two studies [7, 35] examined the association between husband who can read and write with dietary diversity. Unfortunately, in this review, the association between a husband who can read and writes with dietary diversity during pregnancy was statistically insignificant. In addition, three studies [7, 35, 49] assessed husband educated primary and above with dietary diversified food practice. We observe that a woman who had primary and above educated husband had no statistically significant practice of diversified dietary food consumption during pregnancy (Figs 9 and 10).

#### Place of residence

**Fig 11:**
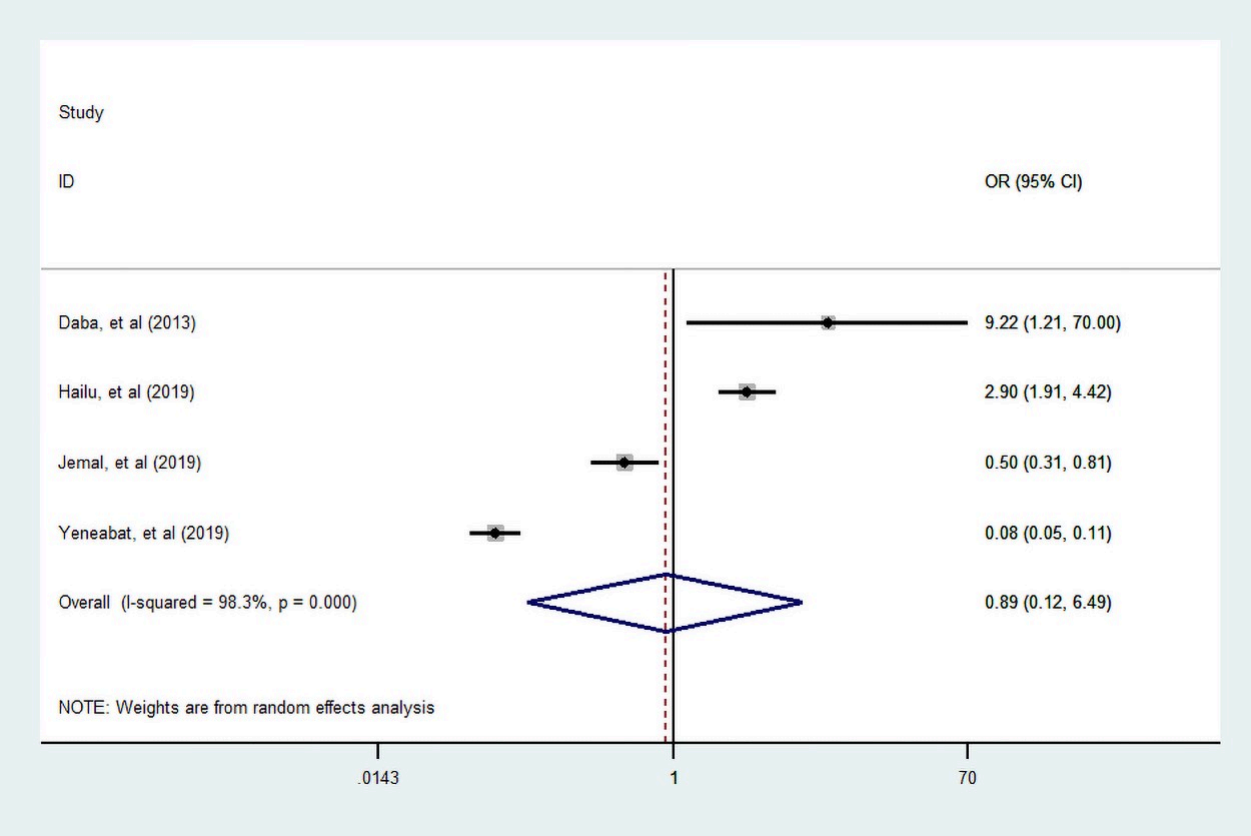
The pooled odds ratio of the association between place of residence and dietary diversity during pregnancy.

In this meta-analysis, four studies [36, 42, 44, 49] were assessed for the association between place of residence and dietary diversity food consumption. Consequently, the association was not statistically significant; it revealed that there is no dietary diversity practice variation among a woman living in rural and urban during pregnancy (Fig 11).

#### Family size

**Fig 12:**
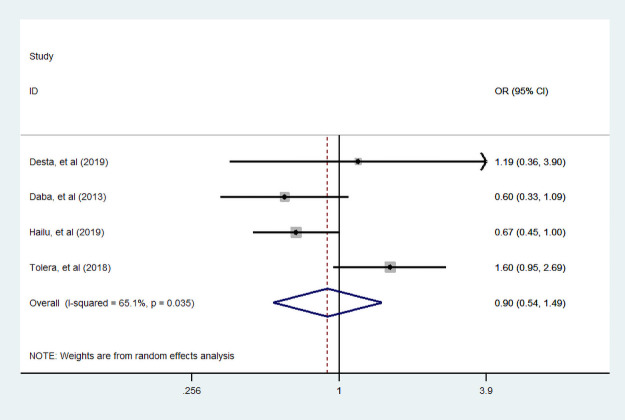
The pooled odds ratio of the association between family size and dietary diversity during pregnancy.

We assessed the association between family size and dietary practice during pregnancy. Four studies [7, 36, 39, 42] assessed its association. Only two studies showed a woman who had more than five family sizes is positively associated with diversified food practice. Our finding indicated that dietary diversity practice during pregnancy was not statistically associated with family size (Fig 12).

#### Nutritional information

**Fig 13:**
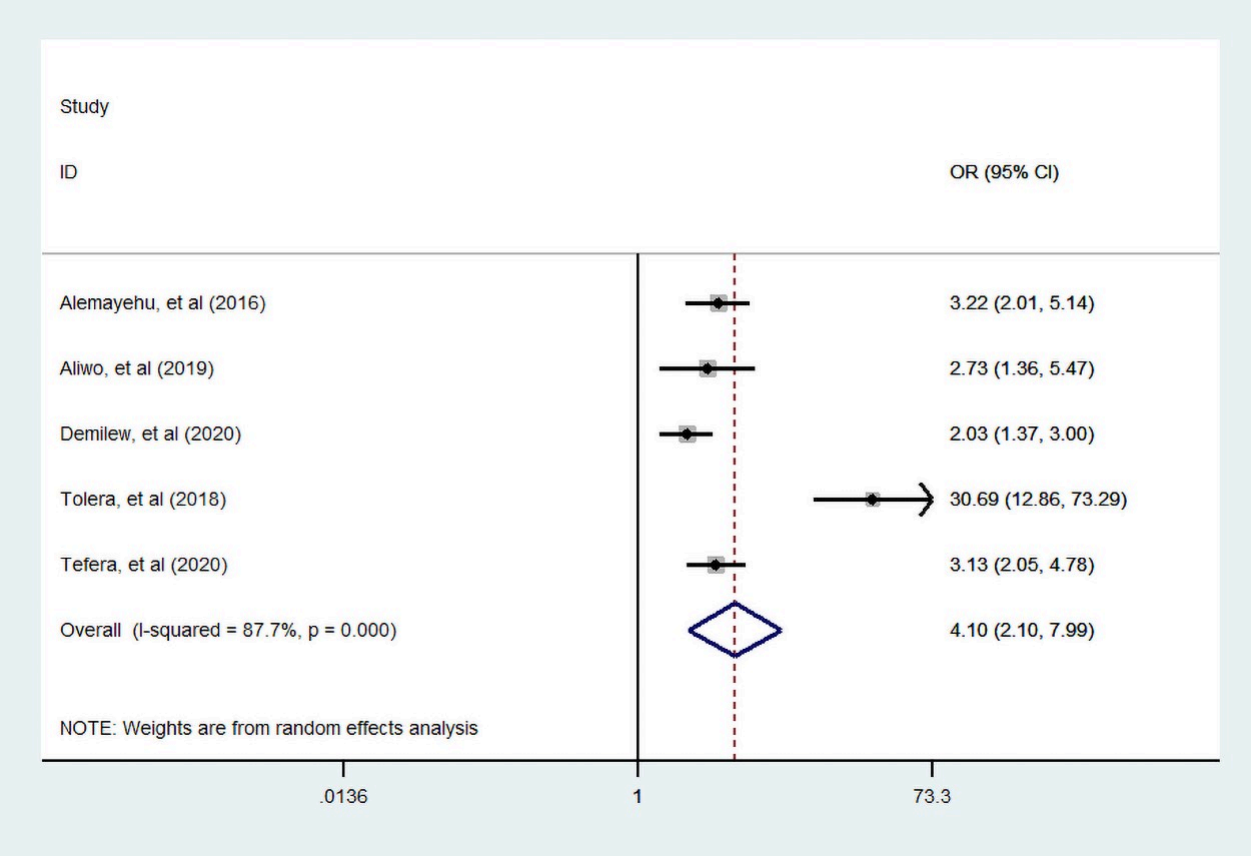
The pooled odds ratio of the association between maternal nutritional information and dietary diversity during pregnancy.

Furthermore, five studies [7, 34, 35, 38, 48] assessed the association between dietary diversity food and nutritional information. We found that statistically significant association between nutritional information and dietary diversity practice during pregnancy. A woman who had nutritional information was 4.1 times more likely practiced diversified food compared with a woman who had no nutritional information (Fig 13).

#### Knowledge about food diversity

**Fig 14:**
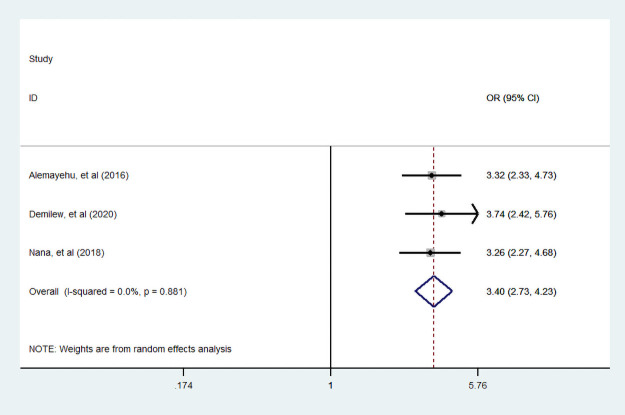
The pooled odds ratio of the association between knowledge about food diversity and dietary diversity during pregnancy.

In three studies [5, 34, 38], investigators examined the association between dietary diversity food knowledge with dietary diversity practice. We found that there was statistically significant association dietary diversity food knowledge and dietary diversity practice. A woman who had good knowledge about dietary diversity food consumption was 3.4 (95% CI: 2.73, 4.73) times more likely to experienced diversified food than who had no knowledge counter parts (Fig 14).

#### Income

**Fig 15:**
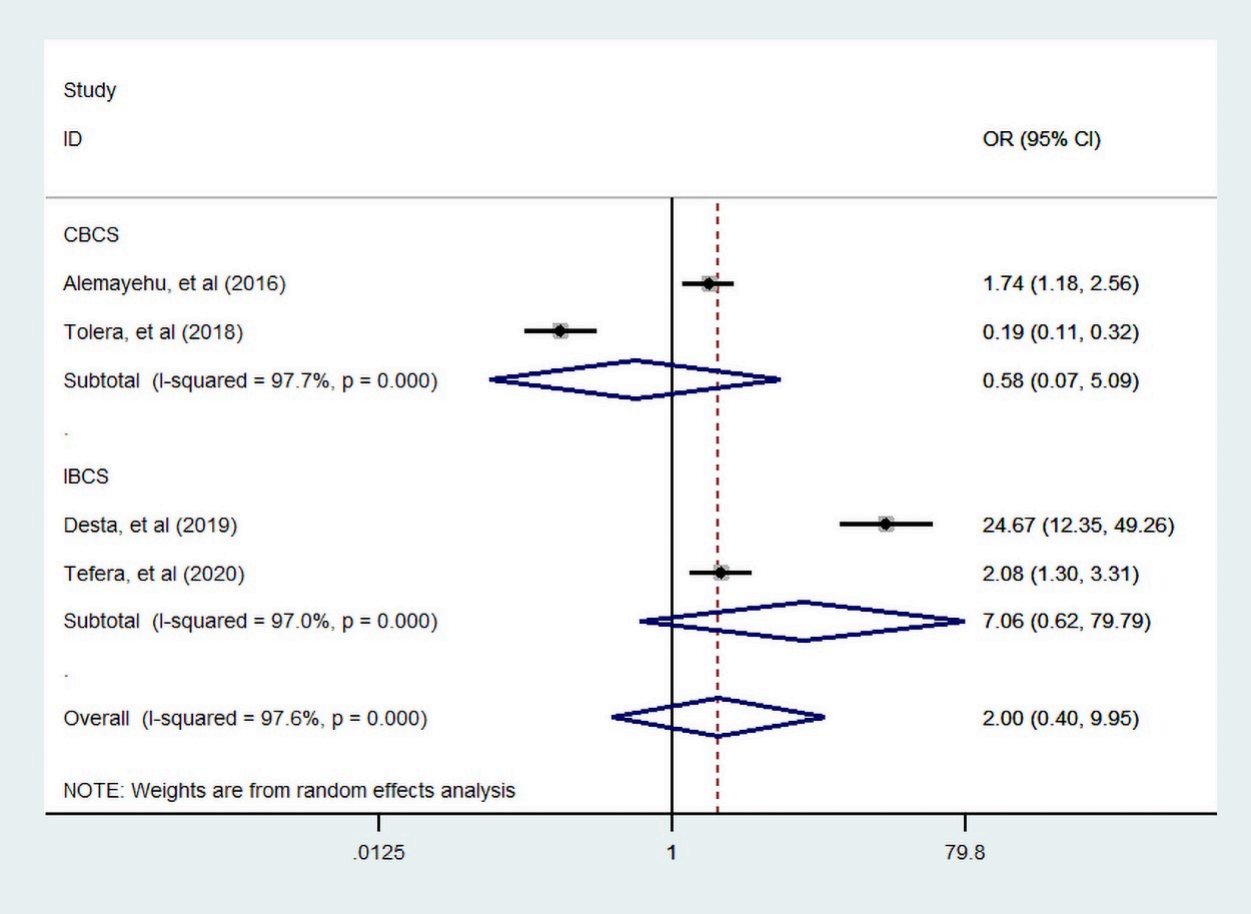
The pooled odds ratio of the association between income and dietary diversity during pregnancy.

In four articles [7, 34, 39, 48], researchers assessed the association of income with dietary diversity practice. Of those studies, two studies were conducted by CBCS. Moreover, one studies reported that income were negative association with dietary diversity during pregnancy. This meta-analysis suggested that income was not statistically associated with dietary diversified practice during pregnancy (Fig 15).

#### Wealth index

**Fig 16:**
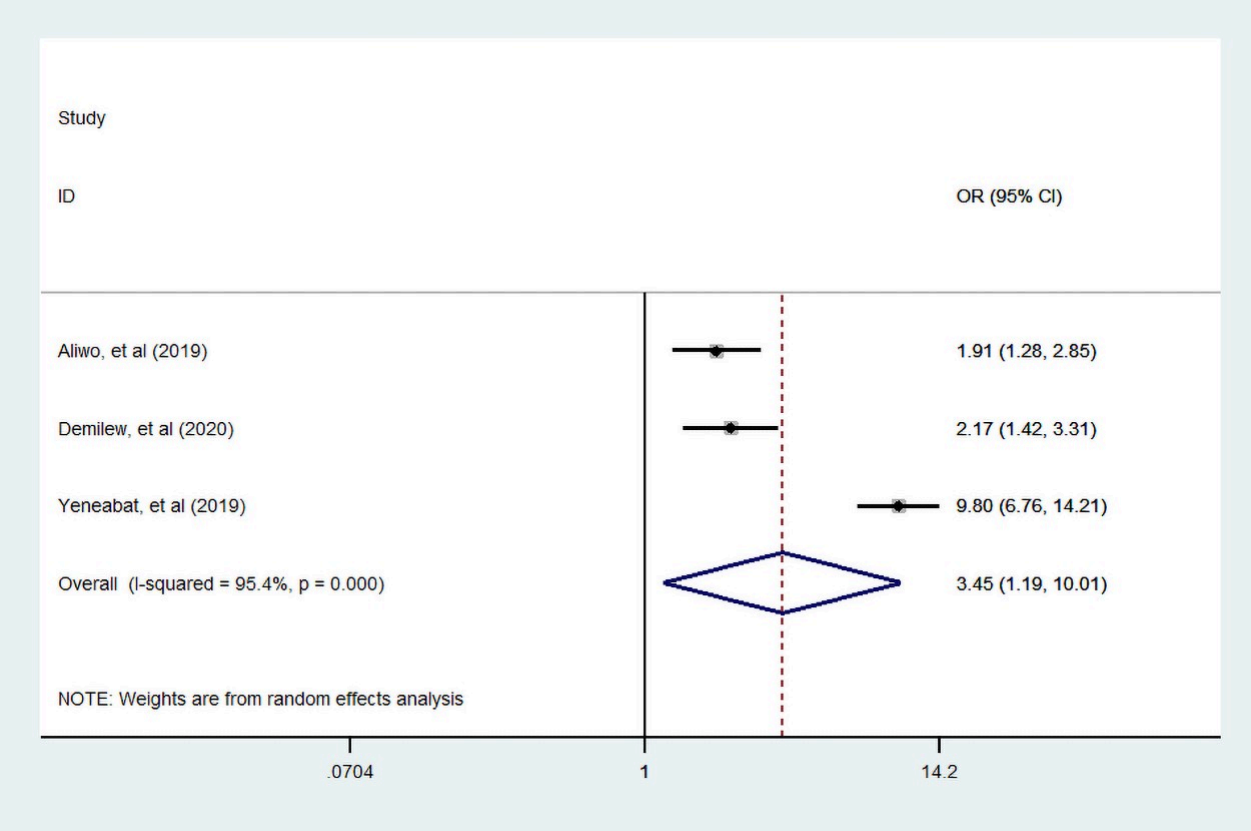
The pooled odds ratio of the association between household high wealth index and dietary diversity during pregnancy.

**Fig 17:**
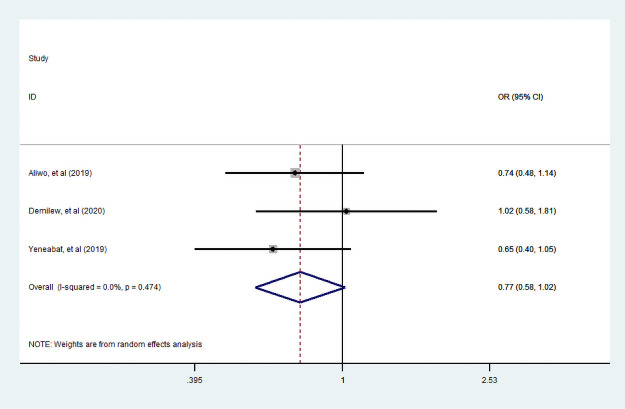
The pooled odds ratio of the association between household medium wealth index and dietary diversity during pregnancy.

Three studies [35, 38, 49] were examined the association between household wealth index and dietary diversity practice. Accordingly those studies, we find that a woman from a household who had high wealth index was more likely practiced dietary diversity food compared to poor household. But, we found that a woman from a household with medium wealth index was not associated with dietary diversity practice compared to poor household (Figs 16 and 17).

## Discussion

Dietary diversity has a significant contribution for pregnancy outcome. Dietary diversity during pregnancy in developing countries is influenced by socio-demographic, culture, individual and health service-related factors [52, 53]. Therefore, this systematic review and meta-analysis was conducted to estimate the pooled proportion and associated factors of dietary diversity practice during pregnancy in Ethiopia.

This meta-analysis showed that the pooled proportion of dietary diversity among pregnant woman in Ethiopia was 41% (95% CI: 33, 49). This finding is almost half of the proportion dietary diversity reported from in Ghana [54], which was a minimum dietary diversity proportion five and above out of 11 food groups was 85% among pregnant woman. Moreover, this result also twice less than the result of a study conducted in Kenya [55], which reported that 85% of pregnant woman consumed more than four varieties of foods. The finding of this study is supported by a study conducted on Zinc deficiency in Ethiopia, which revealed that 59.9% women were faced zinc deficiency during pregnancy, which shows inadequate dietary diversity is high.

In addition, the subgroup analysis indicated that community based cross-sectional studies had (43.3%) more consumed variety of food than institution based cross-sectional study (39.7%). Similarly the highest proportion of dietary diversity was recorded in other group (Addis Ababa, Drie Dewa and Tigray) (49.9%) and the smallest was reported in Oromia (34%). This result has been supported by Ethiopian EDHS 2016 report which states that the highest proportion of normal BMI was observed in Addis Ababa and Drie Dewa city administrations (23.1 and 22.0%) respectively(EDHS,2016) [56]. The reason may be related to sampling issue means that studies which conducted in community based was included more representative participant than institution based studies because institution based studies consider only woman who attained at the selected institution unfortunately woman who left from the institution was not represent in the study. In addition woman who lived in Addis Ababa and Drie Dewa is more educated and have good knowledge relative to other regions.

We also assessed associated factors of proportion of dietary diversity practice in Ethiopia. We found that a woman who can read and write were 82% (95% CI: 1.25, 2.64) more likely to eat diversified food than who cannot read and write. In addition, more educated woman 2.11 (95% CI: 1.10, 4.05) times consumed a variety of food than non-educated woman. This finding is in line with a study conducted in Kenya [55], which showed that more educated woman was 2.78 (95% (CI: 1.06, 5.32)) times consumed minimum dietary diversity food than non-educated woman. We found that dietary diversity practice among pregnant woman was the same across residence and family size.

Moreover, the odds of a women having good nutritional information were 4.1 (95% CI: 2.1, 7.99) times than the odds of women hadn’t information. We also found that a women having knowledge about dietary diversity was 3.4 times more likely practiced diversified food during pregnancy. When women had information and knowledge on nutritional diversity they might have a chance to understand the benefits of consuming diversified food during pregnancy for their own babies’ health. In convention mothers are very egger to the better good of their babies which induces them to practice diversified food. However, husband education does not have significant association with wife’s dietary food consumption during pregnancy. Hence, husband should advise their wife’s to enhance mothers diversity food consumption practice during pregnancy.

Furthermore, this study found that a woman from a rich household was 3.45 (95% CI: 1.19, 10.1) times more likely consumed more diversified food groups compared to a woman from poor household. This finding consistent with a study conducted in Kenya [55], which reported that a women belongs in high income household 2.08 time more consumed a variety of food groups. Studies explain the reason could be related with rich households would have an access variety of foods in the household and it had positive contribution diversified food consumption [57].

## Limitation of the study

This meta-analysis has some limitations. The first limitation of this study was solely studies written in English language were considered to estimate the proportion of dietary diversity among pregnant woman in Ethiopia. In addition, there were not enough studies of systematic reviews and meta-analysis for the problem to compare. Lastly, in this meta-analysis study only four Ethiopian regions and two administrative city of the country were represented, which may reflect non representation due to the limited number of articles included.

## Conclusion

In this meta-analysis; we found that low proportion of dietary diversity among pregnant women in Ethiopia. Maternal education, nutritional information, dietary diversity knowledge and wealth index level of household were significantly associated with dietary diversity practice. Regardless of the current effort towards maternal and child health, this too low proportion of dietary diversity among pregnant women is not acceptable. This study implies the needs to develop interventions to improve house hold wealth by empowering women and improving awareness towards dietary diversity practice during pregnancy through exiting maternal health initiative strategies using health developmental army, policymakers, health extension workers and proper advising by health professionals during antenatal care visits. In addition, Husband education does not help wife’s dietary diversity practice. Hence, husband should advise their wife’s to enhance mothers diversity food consumption practice during pregnancy. Future studies should be given attention on clinical factors like nutritional consult and trimester of ANC during pregnancy.

## Supporting information

S1 Checklist (DOC)

S1 Table Risk of bias assessment. (XLSX)

S2 Table Dietary diversity dataset. (XLSX)

S1 File List of searched articles from electronic database. (TXT)
